# TT Tracker app aims to improve surgical outcomes and patient care

**Published:** 2019-02-10

**Authors:** Kimberly Jensen, Sarah Bartlett, Tim Jesudason

**Affiliations:** 1 Technical Advisor: Global Patient Monitoring, Sightsavers.; 2 Director: mHealth and Innovation, Sightsavers.; 3 Director: Special Projects and Campaign Partnerships, International Coalition for Trachoma Control.

**Trachomatous trichiasis (TT) is a painful and blinding condition in which the eyelashes turn inwards and damage the cornea. TT surgery is the remedy.**

In 2017, the global trachoma elimination programme conducted more than 230,000 operations across 35 countries to treat trachomatous trichiasis (TT), the late blinding stage of trachoma.^1^ TT operations are generally conducted in remote and resource-poor settings, which can make it difficult to follow up and assess surgical outcomes.

The World Health Organization recognised the challenge and convened a meeting in 2015 to discuss the development of a system to track TT patients through the steps of surgical intervention.^2^ In response, Sightsavers developed an Android-based application, called the TT Tracker, which helps surgeons, assistants and supervisors to collect and analyse information about surgical outcomes and performance, and to determine when and where follow-up appointments are required.

Surgical teams using the TT Tracker enter patient information into electronic forms at each stage in the patient's journey, which reduces the need to collect and file paper-based forms. The information collected includes:

- **Registration and evaluation.** Demographic information, TT diagnosis, recommended intervention
- **Surgery.** Type of operation/sutures, name of surgeon, related complications
- **Follow-up (at 24-hours, 7–14 days, and 3–6 months).** Surgical outcome assessment and actions required to address complications

**Figure:**
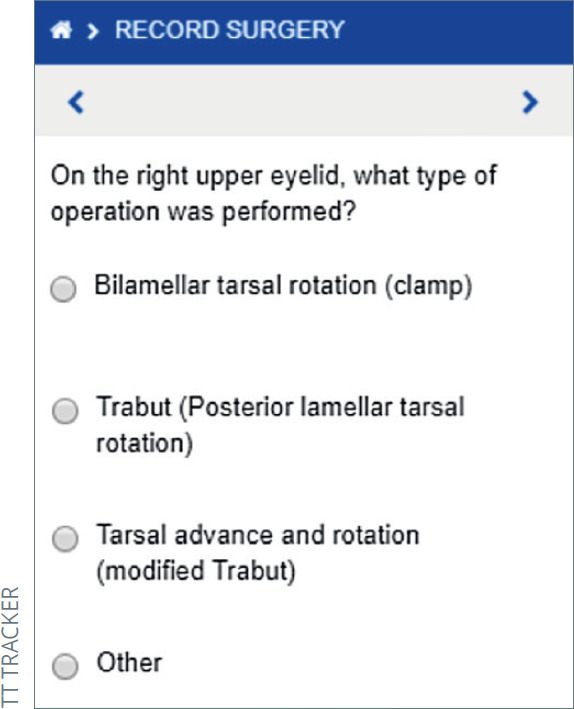
Using TT Tracker to input surgical data.

Common details related to surgical provision, such as the type of suture used, are included in the predefined list of selections (adapted for each country); these are based on the International Coalition for Trachoma Control (ICTC) ‘Organizing trichiasis surgical outreach’ preferred practice manual.^3^ Users may choose to enter additional information if the predefined list is insufficient.

All data are owned by the government and collected on password-protected devices. Surgeons are granted access to patient information within their ministry-designated working area only.

## Data use and access

TT Tracker uses the information collected by surgical teams to generate the following:

**Patient follow-up lists.** After surgery, all patients are automatically placed on follow-up lists based on the relevant follow-up time-period; lists can be accessed by programme staff and officials approved by the Ministry of Health. Reports are electronically sent to supervisors detailing where and when follow-up must take place. Surgical outcomes are assessed during follow-up visits and are added to patient records to ensure each surgical record is complete.

**Figure:**
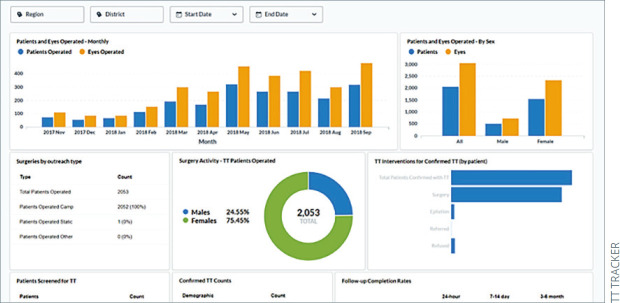
TT Tracker app reports are updated daily.

**Surgical performance evaluation.** The TT Tracker creates performance assessments for individual surgeons so supervisors know when enhanced supervision or additional training is needed. To provide encouragement, surgeons also receive personalised emailed reports including their individual outcomes and contribution to the national elimination effort.

**Timely, easy-to-access reports.** The TT Tracker includes a reporting tool that analyses data automatically. Reports are updated daily and are easy to understand, allowing programme staff to use the data for decision making and to improve the programme, which in turn encourages regular use.

## Rollout and potential for other uses

All national trachoma programmes are eligible to use the TT Tracker to support elimination activities; however, programmes must cover implementation costs, including training, phones and outreach. The tool has been successfully piloted in Sightsavers programmes in Nigeria and Tanzania in 2017/2018 as part of the DFID SAFE programme, followed by a country-wide roll-out in Uganda in July 2018 as part of The Queen Elizabeth Diamond Jubilee Trust's Trachoma Initiative. Full expansion is planned in 2019 in Benin, Guinea, Nigeria, Senegal, Tanzania, and Zimbabwe in the new Accelerate Trachoma Elimination Programme.

As more countries show positive results using the TT Tracker, there is potential to apply it to other surgical eye interventions or other neglected tropical diseases, further contributing to global targets for universal eye health coverage and the UN sustainable development goals.

For more information about the TT Tracker or to get in touch with the team, please visit: **www.tttracker.org/home**
